# Lymphome prostatique: une cause rare de rétention urinaire (à propos d’un cas)

**DOI:** 10.11604/pamj.2022.43.33.33908

**Published:** 2022-09-20

**Authors:** Rabia Yasmine Namaoui, Mohamed Cherif Benremouga, Saï Boutoura, Mohamed Cherif Rahali, Wissam Hadjaoui, Asma Zerabib, Leila Belbekri, Fadéla Zar, Hemana Berrah, Ali Mouats, Abdessamed Bessaïh

**Affiliations:** 1 Service d'Anatomie et Cytologie Pathologiques, Centre de Lutte Contre le Cancer (CLCC) Béchar, Béchar, Algérie,; 2 Service d'Anatomie et Cytologie Pathologiques, Hôpital Central de l´Armée Docteur Mohamed Seghir Nekkache, Alger, Algérie,; 3 Service Hématologie, Hôpital Central de l´Armée Docteur Mohamed Seghir Nekkache, Alger, Algérie,; 4 Université de Béchar Tahri Mohammed, Bechar, Algérie

**Keywords:** Hyperplasie prostatique, lymphome prostatique, prostatisme, cas clinique, Prostatic hyperplasia, prostatic lymphoma, prostatism, case report

## Abstract

Les lymphomes prostatiques (LP) sont rares, moins de 300 cas ont été rapportés dans la littérature. Ils représentent à chaque fois une surprise diagnostique comme dans notre observation qui est typique, didactique et correspond au treizième cas de lymphome prostatique de la zone marginale. Nous rapportons ici, le cas d'un patient âgé de 65 ans, sans antécédent, ayant présenté des signes de prostatisme durant 4 mois, aggravés par la survenue d'une rétention urinaire aiguë. Le diagnostic d'hyperplasie bénigne prostatique avec un taux de prostate-specific antigen (PSA) normal a été posé aisément et le patient a bénéficié d'une résection transurétrale. A notre grande surprise, l'étude histologique a mis en évidence une infiltration prostatique massive par un lymphome non hodgkinien (LNH) de type zone marginale (MALT), le bilan d'extension l'a classé selon Steuter comme un lymphome prostatique de stade IV B avec conversion leucémique. Le patient est actuellement en rémission depuis 18 mois avec normalisation des lactate déshydrogénase (LDH) après introduction d'un traitement de type R-chop. Bien que rares, ces localisations ne doivent pas être omises, un dosage des LDH doit être systématique devant des signes prostatiques et un taux de PSA normal. Le pronostic est variable, lié à l´âge, au type histologique et au stade évolutif, cependant, la médiane de survie est identique pour les formes primitives et secondaires.

## Abstract (en)

Prostate lymphomas (LP) are rare, fewer than 300 cases have been reported in the literature. Each time they are a diagnostic surprise as is the case with our study that is typical, didactic and reports the thirteenth case of prostatic lymphoma in the marginal zone. The study involved a 65-year-old patient, with no particular previous history who presented with signs of prostatism lasting for 4 months, aggravated by the occurrence of acute urinary retention. The diagnosis of benign prostatic hyperplasia with normal prostate-specific antigen (PSA) level was easily made and the patient underwent transurethral resection. To our surprise, the histological study revealed a massive infiltration of prostatic tissue by a non-Hodgkin lymphoma (NHL), marginal zone lymphoma (MZL) subtype. According to Steuter, it was classified as prostatic lymphoma stage IVB with leukemic transformation. The patient has experienced remission for 18 months with normalization of LDH after R-chop therapy. Although rare, these sites of occurrence should be suspected, LDH assay should be systematic in patients with prostatic signs and normal PSA levels. Prognosis is variable, according to age, histologic type and evolutionary stage, however, the median overall survival is identical for primary and secondary forms.

## Introduction

Les lymphomes sont des proliférations malignes pouvant être d'origine ganglionnaire ou extra-ganglionnaire; moins de 10% intéressent l'arbre urinaire et l'atteinte prostatique est rare, moins de 300 cas ont été rapportés dans la littérature, dont la majorité étaient des formes secondaires [1]. Les localisations primitives prostatiques représentent 0.2 à 0.8% des lymphomes extra ganglionnaires et sont définies selon Bostwick comme une localisation prostatique exclusive sans atteinte ganglionnaire, hépatique, splénique ou médullaire durant au moins un mois du diagnostic [1,2]. Néanmoins pour Steuter, une symptomatologie prostatique initiale isolée suffit à ce diagnostic indépendamment du stade évolutif [3] comme dans notre observation qui est didactique et représente le treizième cas d'un lymphome de la zone marginale prostatique rapporté.

## Patient et observation

**Présentation du patient:** il s'agit d'un patient de 65 ans, sans antécédent, ayant présenté durant 4 mois une dysurie avec pollakiurie qui s'est compliquée d'une rétention urinaire aigue, motivant une consultation aux urgences.

**Résultats cliniques:** l'examen clinique a retrouvé un patient en bon état général, présentant un globe vésical douloureux et une volumineuse masse prostatique ferme et homogène au touché rectal. Le reste de l'examen était sans particularité notamment l'exploration des aires ganglionnaires superficielles. Devant ce tableau clinique une échographie abdomino-pelvienne, un bilan standard et un dosage du taux de PSA ont été réalisés en urgence.

**Démarche diagnostique:** l'exploration échographique abdomino-pelvienne réalisée n'a retrouvé que des signes évocateurs d'une hyperplasie bénigne prostatique (HBP) simple sans mise en évidence d'hydronéphrose, d'adénopathie ou d'hépato-splénomégalie. Le dosage du taux de PSA était normal et le reste du bilan standard et préopératoire étaient sans particularité.

**Intervention thérapeutique:** devant ce tableau de rétention urinaire aigue sur HBP, le patient a bénéficié d'un sondage urinaire afin de le soulager dans l'immédiat suivi par la suite d'une résection prostatique endoscopique transurétrale permettant un traitement au long terme. Les suites opératoires étaient simples avec amélioration de la symptomatologie.

**Figure 1:**
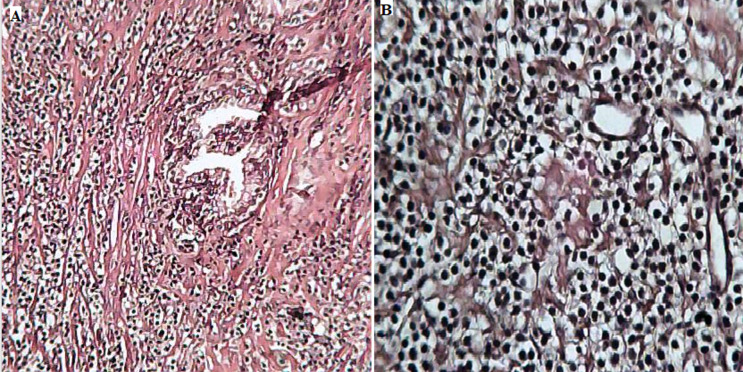
A) parenchyme prostatique avec coulées de petits lymphocytes; B) éléments monomorphes et d'aspect monocytoïde

**Figure 2:**
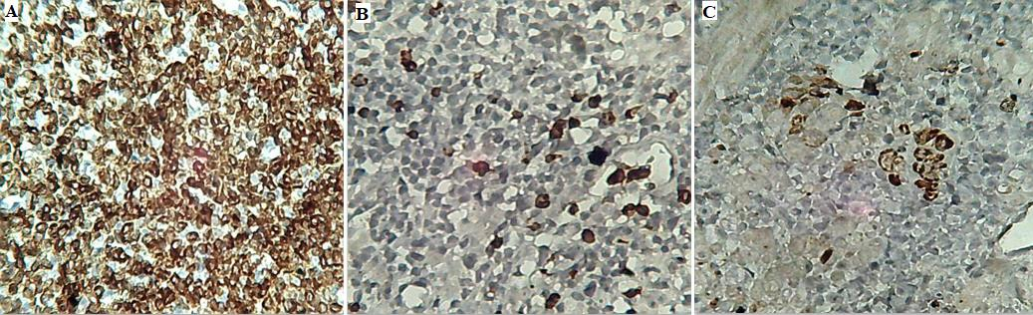
étude immunohistochimique: A) nappes de petits éléments lymphoïdes B CD20+; B) présence de quelques lymphocytes T réactionnels CD3+; C) reliquats de glandes détruites et grignotées marquées par la panCK

**Résultat et suivi:** à notre grande surprise l'étude histologique des copeaux de résection prostatiques adressés a mis en évidence un parenchyme prostatique largement infiltré par des coulées de lymphocytes de petite taille, d'aspect monocytoïde et centrocytique, montrant par places des images de lymphocytose et de destructions lympho-épithéliales (Figure 1). Ces éléments étaient de phénotype B: CD20+, CD79a+, CD3-, CD5-, CD23-, Cycline-D1- et CD10- avec un faible indice de prolifération Ki67, estimé à 1% (Figure 2). Ainsi, le diagnostic d'un lymphome prostatique à petites cellules B de la zone marginale extra-ganglionnaire de type MALT a été posé.

Le bilan d'extension a retrouvé un discret amaigrissement récent, des adénopathies cervicales, iliaques et une hépato-splénomégalie minimes; un taux des LDH modérément élevé, un pic monoclonal kappa et une lymphocytose sanguine sans infiltration médullaire dont le profil en cytométrie de flux était similaire à celui de l'infiltrat prostatique, classant ainsi ce lymphome en lymphome prostatique de la zone marginale (type MALT) de stade IV-B d´Ann Arbor en conversion leucémique. Le bilan virologique a mis en évidence par ailleurs, une hépatite B chronique active concomitante. Le patient a bénéficié d'une polychimiothérapie de type R-CHOP ayant permis une rémission complète à 3 mois avec normalisation des LDH et une survie sans rechute à 18 mois.

**Consentement éclairé:** après des explications claires et détaillées, le patient a donné son consentement éclairé vu qu'il est majeur pour la réalisation de ce travail.

## Discussion

Les localisations lymphomateuses prostatiques sont rares et représentent 0.09% des néoplasmes prostatiques. Seulement 35% sont primitifs; ce qui implique une localisation prostatique exclusive durant au moins un mois du diagnostic [1,2]. Cependant pour Steuter, une symptomatologie initiale prostatique isolée suffit au diagnostic indépendamment du stade évolutif [3] comme dans notre observation. Les LP surviendraient à la sixième décade, essentiellement chez les immunodéprimés ou porteurs d'une infection virale *Epstein-Barr virus* (EBV).

Les signes de prostatisme sont au premier plan, leur installation rapide associée à l'augmentation des LDH sériques doit alerter car le taux de PSA est souvent normal. Le toucher rectal, la cystoscopie, l'échographie prostatique et la tomodensitométrie (TDM) pelvienne ne peuvent les différencier d'une HBP [4]. En cas de doute, le *positron emission tomography* (PET) scan à la 18F-fluorodeoxyglucose (^18^FFDG) serait utile pour soulever le diagnostic de malignité mais aussi pour apprécier le bilan d'extension et le suivi thérapeutique.

Seule l'analyse des copeaux de résection ou des biopsies permet le diagnostic. La majorité des LP sont des LNH-B, souvent de haut grade à grandes cellules; ou bien indolents à petites cellules sauf pour ceux du manteau qui sont agressifs. L'association à un adénocarcinome est possible [2,4]. Notre observation est la treizième localisation prostatique d'un lymphome de la zone marginale extra-ganglionnaire rapportée. Son diagnostic différentiel se pose essentiellement avec une prostatite chronique mais le caractère diffus, destructeur et monotypique de l'infiltrat l'élimine rapidement.

La rareté des publications explique l´absence d'un consensus thérapeutique. La chimiothérapie reste le traitement de choix, essentiellement depuis l'adjonction du rétuximab pour les LNH-B qui a nettement amélioré la survie. Le traitement peut être associé à une radiothérapie ou une chirurgie afin d'améliorer la symptomatologie [1-4]. Le pronostic est variable mais la médiane de survie est similaire pour les formes primitives et secondaires. Le risque de rechute est d'autant plus élevé que les facteurs de mauvais pronostic coexistent et qui sont: l´âge supérieur à 60 ans, l'élévation du taux sérique des LDH, les stades avancés (III et IV) et les formes histologiques agressives [1,2].

## Conclusion

Le LP dont l´expression clinique ne diffère guère de la symptomatologie classique de l´HBP est une pathologie rare. Son mauvais pronostic est autant lié aux formes histologiques de haut grade qu´au diagnostic souvent tardif. Mais depuis l'administration du retuximab en association à la chimiothérapie conventionnelle pour les lymphomes B, la survie s'est nettement améliorée.

## Remerciements

Remerciements et hommage au Docteur Ahmed Kacimi pour son parrainage et ses apprentissages au cours des années travaillées au sein de son service.
